# Regorafenib Effects on Human Colon Carcinoma Xenografts Monitored by Dynamic Contrast-Enhanced Computed Tomography with Immunohistochemical Validation

**DOI:** 10.1371/journal.pone.0076009

**Published:** 2013-09-30

**Authors:** Clemens C. Cyran, Philipp M. Kazmierczak, Heidrun Hirner, Matthias Moser, Michael Ingrisch, Lukas Havla, Alexandra Michels, Ralf Eschbach, Bettina Schwarz, Maximilian F. Reiser, Christiane J. Bruns, Konstantin Nikolaou

**Affiliations:** 1 Department of Clinical Radiology, Laboratory for Experimental Radiology, University Hospitals Munich, Grosshadern Campus, Muenchen, Germany; 2 Department of Clinical Radiology, Josef-Lissner-Laboratory for Biomedical Imaging, University Hospitals Munich, Grosshadern Campus, Muenchen, Germany; 3 Department of Surgery, University Hospitals Munich, Grosshadern Campus, Muenchen, Germany; Technische Universität München, Germany

## Abstract

**Objective:**

To investigate dynamic contrast-enhanced computed tomography for monitoring the effects of regorafenib on experimental colon carcinomas in rats by quantitative assessments of tumor microcirculation parameters with immunohistochemical validation.

**Materials and Methods:**

Colon carcinoma xenografts (HT-29) implanted subcutaneously in female athymic rats (n = 15) were imaged at baseline and after a one-week treatment with regorafenib by dynamic contrast-enhanced computed tomography (128-slice dual-source computed tomography). The therapy group (n = 7) received regorafenib daily (10 mg/kg bodyweight). Quantitative parameters of tumor microcirculation (plasma flow, mL/100 mL/min), endothelial permeability (PS, mL/100 mL/min), and tumor vascularity (plasma volume, %) were calculated using a 2-compartment uptake model. Dynamic contrast-enhanced computed tomography parameters were validated with immunohistochemical assessments of tumor microvascular density (CD-31), tumor cell apoptosis (TUNEL), and proliferation (Ki-67).

**Results:**

Regorafenib suppressed tumor vascularity (15.7±5.3 to 5.5±3.5%; p<0.05) and tumor perfusion (12.8±2.3 to 8.8±2.9 mL/100 mL/min; p = 0.063). Significantly lower microvascular density was observed in the therapy group (CD-31; 48±10 vs. 113±25, p<0.05). In regorafenib-treated tumors, significantly more apoptotic cells (TUNEL; 11844±2927 vs. 5097±3463, p<0.05) were observed. Dynamic contrast-enhanced computed tomography tumor perfusion and tumor vascularity correlated significantly (p<0.05) with microvascular density (CD-31; r = 0.84 and 0.66) and inversely with apoptosis (TUNEL; r = −0.66 and −0.71).

**Conclusions:**

Regorafenib significantly suppressed tumor vascularity (plasma volume) quantified by dynamic contrast-enhanced computed tomography in experimental colon carcinomas in rats with good-to-moderate correlations to an immunohistochemical gold standard. Tumor response biomarkers assessed by dynamic contrast-enhanced computed tomography may be a promising future approach to a more personalized and targeted cancer therapy.

## Introduction

In recent years, a variety of novel molecular cancer therapies have been introduced into clinical routine. Amongst others, anti-angiogenic strategies, such as tyrosine kinase inhibitors and monoclonal antibodies, have shown their potential as effective treatment options in different malignancies [1]–[3]. Contrast-enhanced computed tomography (CE-CT) is a widely used tool for the clinical evaluation of the effect of anti-cancer therapy, using established morphology-based imaging criteria, such as the Response Evaluation Criteria in Solid Tumors (RECIST) [4], [5]. However, it has become clear that morphology-based CE-CT alone is not sufficient for early monitoring of the therapeutic effect of molecular anti-cancer agents, as these treatment regimens are not primarily cytotoxic [6]. The regimens do have significant impact on tumor angiogenesis and metabolism, but may have only subtle effects on tumor morphology, especially during initial treatment [7]. Consequently, there is a rising demand for non-invasive imaging biomarkers that allow for early monitoring of therapeutic effects on tumors *in vivo* [8]. In the long term, these biomarkers could contribute to a multimodal oncologic therapy concept, facilitating the management of a personalized and more targeted cancer treatment [9].

Various dynamic contrast-enhanced (DCE) imaging modalities, such as CT, magnetic resonance imaging (MRI), and ultrasound, allow for the non-invasive imaging of tumor microcirculation [10]–[13]. DCE-CT has been used to quantify tumor perfusion, blood volume, and vascular permeability, parameters which often are pathologically increased in malignant tumors [12]. Several previous trials have demonstrated that tumor blood flow and volume, as depicted by DCE-CT allow for the monitoring of anti-angiogenic therapy in renal [14], [15], lung [16], prostate [17], and colorectal cancer [18], [19]. Regorafenib is a novel oral multi-kinase inhibitor which exhibits anti-angiogenic and anti-proliferative effects on glioblastoma, breast, and renal cell carcinoma xenografts *in vivo* [20]. It has been shown to be effective in metastastic colorectal cancer [21].

We therefore hypothesized that DCE-CT could be used for the non-invasive monitoring of anti-angiogenic and anti-proliferative effects of regorafenib in a subcutaneous model of human colorectal carcinoma in rats. The purpose of our study was to investigate functional parameters of tumor microcirculation assessed by DCE-CT as non-invasive biomarkers for the effect of regorafenib monotherapy on experimental human colorectal carcinoma xenografts using immunohistochemical validation.

## Materials and Methods

### Animal Model

The study was approved by the Government of Upper Bavaria Committee for Animal Research (Gz.55.2-1-54-2532-33-10) and was carried out in accordance with the guidelines of the National Institute of Health for the care and use of laboratory animals. Female athymic rats (n = 15, 7–8 weeks old, Harlan Laboratories Inc., Indianapolis, IN) were injected subcutaneously with 6×10^6^ cells of the human colon carcinoma cell line HT-29 (ATCC HTB-38) suspended in a total volume of 0.5 mL as a 1∶1 mixture of phosphate buffered saline pH 7.4 (PBS) and Matrigel™ (BD Biosciences, San Jose, CA). Subcutaneous xenografts were allowed to grow to a size of ∼800 mm^3^, assessed by daily caliper measurements in three dimensions (a×b×c×0.5). Animals were randomly assigned to either the treatment (n = 7) or to the control group (n = 8). All animals were scanned by DCE–CT on day 0 and day 7 of a one-week daily treatment protocol with the multi-tyrosine kinase inhibitor, regorafenib (Bayer HealthCare, Leverkusen, Germany), or with the placebo (1∶1 solvent solution of cremophor/ethanol). Subsequent to imaging on day 7, animals were euthanized and tumors were explanted, marked at the cranial end of the tumor, fixed in formalin and preserved by cryopreservation for immunohistochemical workup.

### Treatment

The treatment group received 10 mg/kg body weight of regorafenib daily via gastric gavage, using a dedicated 16-gauge curved buttoned cannula. To a vigorously stirred solution of 20 mg regorafenib in 2.5 mL of a 1∶1 solution of cremophor/ethanol at 60°C was added 7.5 mL of distilled water. The control group received volume-equivalent applications of the regorafenib solvent daily.

### DCE-CT Imaging

Prior to CT scanning, animals were anaesthetized by isoflurane (5% for induction, 2.5% for maintenance, administered in pure oxygen). A 25-gauge butterfly catheter (B. Braun AG, Melsungen, Germany) was inserted into a tail vein for contrast injection. Scans were conducted on a clinical 128-slice dual source CT system (Somatom Definition Flash, Siemens Healthcare, Forchheim, Germany) with animals placed in a head-first supine position on the scanner table. Initial scout views and a non-enhanced spiral scan of the animal chest and abdomen were assessed in order to facilitate further planning of the individual scan range for tumor perfusion imaging. The DCE-CT scan interval was 90 seconds, started 5 seconds before contrast injection of 2 mL/kg bodyweight of iopromide (Ultravist 370®, Bayer HealthCare, Berlin, Germany) with a target contrast injection volume of 0.6 mL. Automated bolus injection was performed at an injection rate of 7.2 mL/min using a dedicated automated syringe pump (Harvard Apparatus PHD2000 series, Instech Laboratories Inc., Plymouth Meeting, PA). Detailed scan settings were as follows: scan range 38.4 mm (equals the detector width, no table movement during scan); 80 kV tube voltage; 200 mAs tube current; spatial resolution acquired 0.5×0.5×0.5 mm, in-plane spatial resolution reconstructed 0.13×0.13 mm (matrix, 512×512; field-of-view, 65 mm). Reconstructed slice thickness was 1.0 mm in order to improve the signal-to-noise ratio (SNR) with a total number of 38 reconstructed slices per time-frame. 180 data sets at a temporal resolution of 0.5 s were acquired. A medium soft kernel (B30S) was used for image reconstruction.

### Data Processing and Kinetic Analysis

**Figure 1:**
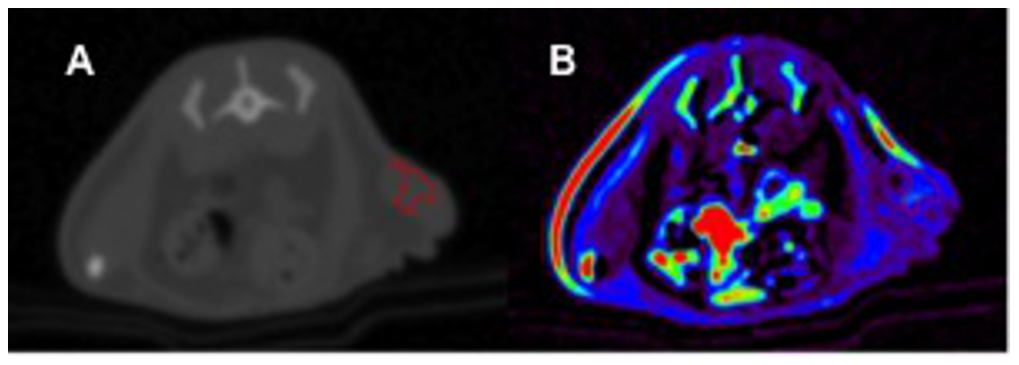
ROI selection. Axial contrast-enhanced CT of an animal after 7 days of regorafenib monotherapy (A). Note the subcutaneous tumor xenograft in the left lateral flank. For measurements of tumor microperfusion, a ROI (red lined area) was drawn using semiquantitative AUC maps (B). Note the right lateral flank colored red due to a skinfold artifact.

Data post-processing was performed at an offline-workstation using PMI software 0.4 (PMI 0.4; Platform for Research in Medical Imaging), written in-house in IDL 6.4 (ITT Visual Information Systems, Boulder, Colorado). Signal enhancement S(t)–S(0) was used for approximation of contrast agent concentration. A region-of-interest (ROI) was drawn in the central lumen of the aorta (4×4 pixels) for acquisition of an arterial input function (AIF). The branch of the renal artery was used as a landmark for a reproducible ROI positioning. To standardize kinetic analysis and to minimize inter-observer bias in the selection of the tumor ROI, semi-quantitative maps served to identify viable tumor tissue (Fig. 1). Area under the curve/maximum of the curve (AUC/max) is a semi-quantitative parameter related to plasma volume and mean transit time. AUC/max was used for ROI definition, as it is a robust parameter and allows for differentiation between presumably vital and necrotic areas of the tumor. High levels of interstitial pressure and necrosis are known to occur in the center of the tumor, resulting in altered contrast media kinetics. Therefore, the tumor ROI was drawn on areas within the tumor periphery which are known to be representative of vital tumor tissue [17]. An area within 5 mm from the cranial or caudal tumor margin was selected for DCE kinetic analysis. The skin was excluded from these measurements. This information also facilitated the choice of immunohistochemical tumor section in order to ensure an approximated match. A two-compartment uptake (2-CU) model was employed for quantification of perfusion and endothelial permeability in these regions [22].

In this model, the tissue concentration c(t) is expressed as the convolution of the arterial concentration c_a_(t) with a two-parameter residue function:
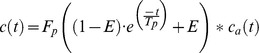
where F_p_ is the plasma flow, T_p_ is the plasma transit time and E is the extraction fraction. Thus, the relative plasma volume V_p_ and the permeability-surface area product PS, i.e, the flow across the capillary wall, can be calculated as:



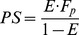
and



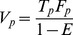



Estimates of F_p_, PS, and V_p_ were provided fitting this model to all tumor curves at an assumed fixed hematocrit value 0.45.

### Immunohistochemistry

The formaldehyde-fixed and paraffin-embedded tissue was stained with focus on various aspects of tumor therapy effects, specifically microvascular density (CD-31), tumor cell proliferation (Ki-67), and cell apoptosis (TUNEL). Tissue samples were de-waxed and re-hydrated following standard procedures (pre-heating at 60°C, washing in xylene substitute [Neo-Clear, Merck KgaA, Darmstadt, Germany], and re-hydration in a graded series of ethanol [100%, 96%, 90% and 70% ethanol] followed by double distilled water). Subsequently, dedicated staining procedures were initiated.

### Ki-67 Antigen Staining

A Ki-67-specific monoclonal rabbit anti-rat antibody was used to quantify tumor cell proliferation. The tissue was demasked in 0.1 M Citrate buffer (pH = 6.0) using microwave irradiation at 600 W. Permeabilization was achieved by immersing the slides in 0.25% Triton X-100 - Tris-Cl solution. After application of the primary antibody (ab16667, 1∶100), a multi-step kit was used for the secondary antibody application following the manufacturer’s instructions (Dako EnVision+ System HRP (DAB), Dako, Germany). Results were quantified as the average number of proliferating cells in 10 random fields at 200×magnification.

### CD-31 Antigen Staining

For immunohistochemical assessment of tumor microvascular density, tumor sections were incubated with the primary antibody (ab28364 1:50) overnight. Further work up of tissue samples was performed using the EnVision+ System HRP (AEC) (DAKO Diagnostika, Germany), and tumor microvessels were quantified as previously described in detail [23]. Results were quantified as the average number of endothelial cells in 10 random fields at 200×magnification.

### TUNEL (Terminal Deoxynucleotidyl Transferase–mediated Nick End Labeling) Staining

TUNEL staining was performed using a commercially available apoptosis detection kit (*in situ* Cell Death Detection Kit, Roche Diagnostics, Indianapolis, IN) according to the manufacturer’s instructions (www.roche-applied.science.com). Samples were subsequently analyzed using a fluorescence microscope and a standard fluorescent filter set at 520±20 nm. Results were quantified as the average number of apoptotic cells in 10 random fields at 200×magnification.

### Statistical Analysis

Continuous variables are presented as means and standard deviations. DCE-CT and immunohistochemistry values between the treatment and the control group were compared using a Mann-Whitney U test. For intragroup comparisons of the DCE-CT perfusion parameters between day 0 and day 7, a Wilcoxon signed-rank test was applied. Analyses were carried out using SPSS for windows (version 11.5, IBM, Armonk, New York). Relationships between DCE-CT and immunohistochemistry were evaluated using Pearson’s correlation coefficients. P-values <0.05 were considered statistically significant.

## Results

DCE-CT and immunohistochemical evaluation of tumors were successfully completed in 15 animals (therapy group, n = 7; control group, n = 8).

### DCE-CT

**Figure 2:**
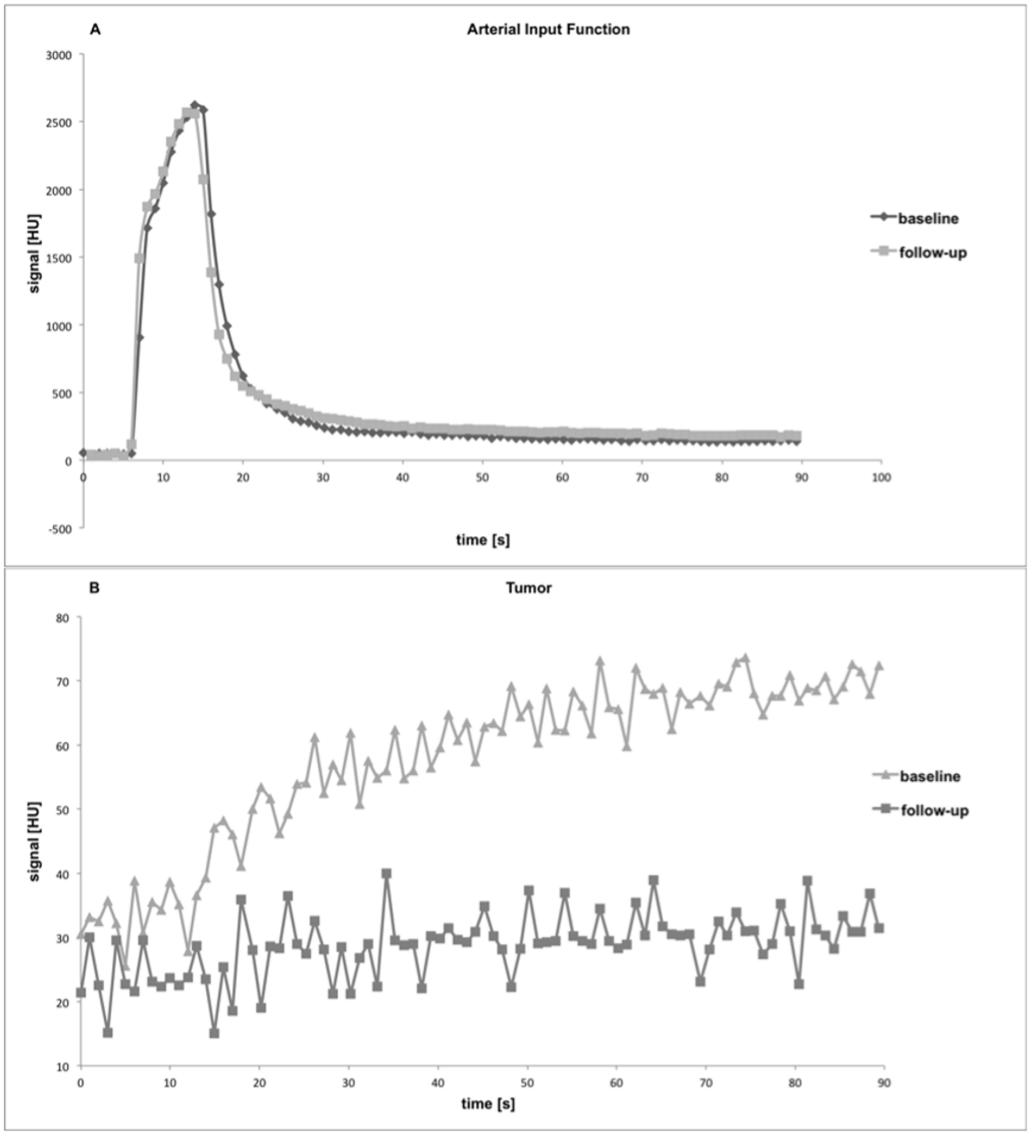
Representative arterial input functions (A) and tumor signal enhancement curves (B) at baseline and follow-up. Signal intensity [HU, Hounsfield Units] (y-axis) is displayed over time [s] (x-axis). Note the almost identical arterial input functions for baseline and follow-up which can be considered a marker of good reproducibility of individual perfusion scans. After anti-angiogenic therapy, a decline in tumor signal enhancement due to reduced tumor perfusion was observed.

**Figure 3:**
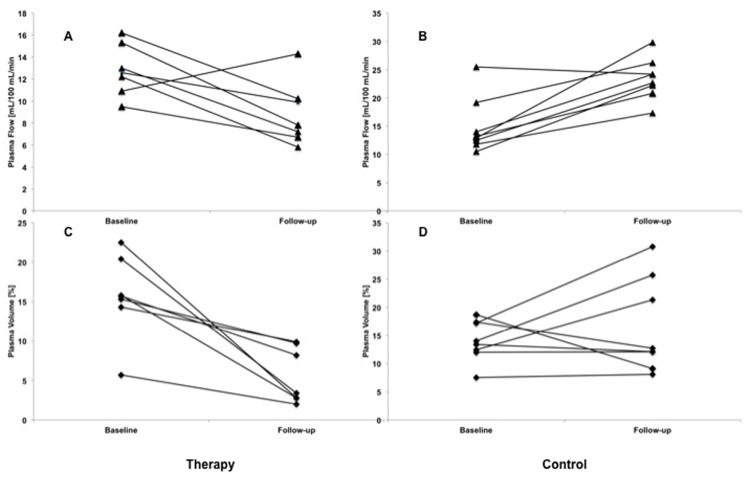
Development of individual values for plasma flow and plasma volume in therapy and control group. Plasma Flow (F_p_) and Plasma Volume (V_p_) at baseline and follow-up are displayed at the y-axis. Note the unidirectional development of F_p_ in both groups (A, B) and of V_p_ in the therapy group (C). However, in one animal from the control group, we observed a decline in V_p,_ which can be attributed to fast tumor growth and a high level of necrosis in the tumor (D).

**Figure 4:**
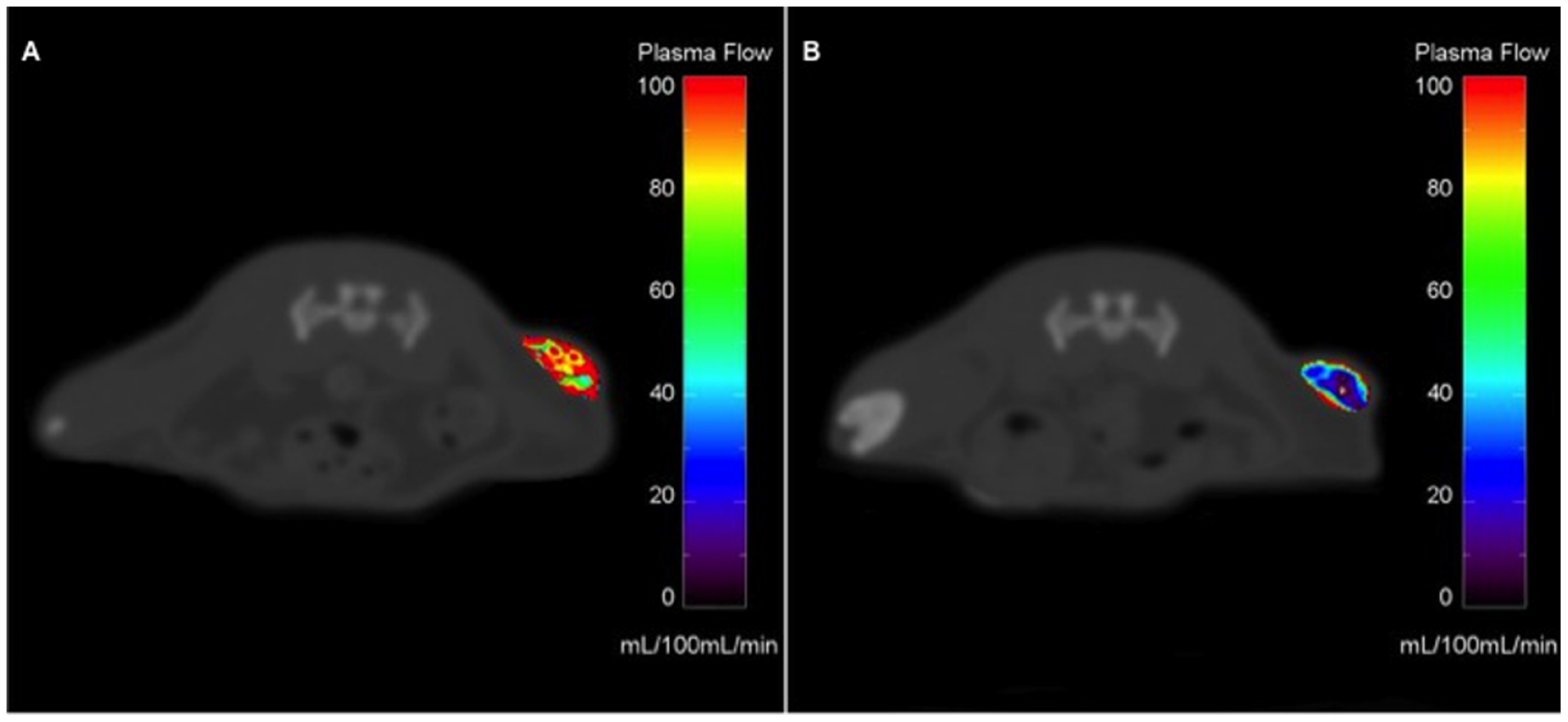
Representative color-coded maps for plasma flow (F_p_) pre (A) and post therapy (B). Plasma Flow (F_p_) values [mL/100 mL/min] are coded according to the vertical color scale. For demonstration purposes only and in contrast to the actual plasma flow assessment, pixel-wise analysis has been conducted here in order to create color-coded maps for a better visualization of the effects of regorafenib therapy. As their position relative to the tumor remains constant, vertebral bodies were used as topographic landmarks to assure analysis of comparable tumor slices. Note the adducted left lower limb in the post therapy image, which does not have an effect on tumor position. Before regorafenib therapy, we observed a highly perfused tumor with a hyperperfused rim (A). After regorafenib therapy, tumor perfusion has declined significantly, predominantly in the center of the tumor (B).

The applied 2-CU model fit the data well in all experiments with a representative best fit and an arterial input function shown in Fig. 2. In the therapy and the control group, significant changes of tumor perfusion were observed between baseline (day 0) and follow-up (day 7). In the therapy group, mean F_p_ declined from 12.8±2.3 at day 0 to 8.8±2.9 mL/100 mL/min at day 7 (p = 0.063) (Fig. 3). In the control group, F_p_ increased significantly from 15.0±5.0 at day 0 to 23.4±3.7 mL/100 mL/min at day 7 (p<0.05). In the therapy group, V_p_ decreased significantly from 15.7±5.3 at day 0 to 5.5±3.5% at day 7 (p<0.05), with a uni-directional decline observed in all animals (Fig. 3). In the control group, V_p_ increased non-significantly from 14.1±3.6 at day 0 to 16.5±8.4% at day 7 (p>0.05). In the therapy group, a non-significant reduction in PS from 7.4±5.8 (baseline) to 3.3±0.9 mL/100 mL/min (day 7) was observed (p>0.05). In the control group, PS showed a significant increase from 4.8±3.6 (day 0) to 9.0±3.2 mL/100 mL/min (day 7) (p<0.05). Individual values for baseline and follow-up measurements in the therapy and the control group are displayed in Tables 1 and 2. A representative image of tumor contrast enhancement at baseline and follow-up is shown in Fig. 4.

**Table 1:** Individual values of functional parameters of tumor microcirculation in the therapy group at baseline and follow-up.

Animal no.	^a^F_p_	^a^V_p_	^a^PS	^b^F_p_	^b^V_p_	^b^PS
1	12.6	15.7	6.8	9.9	8.2	1.9
2	13.0	15.3	3.7	7.2	9.7	4.3
3	12.2	15.8	2.2	5.8	2.8	3.5
4	15.3	22.5	19.4	7.8	2.8	3.0
5	10.9	14.3	4.0	14.3	9.9	4.2
6	9.5	5.7	6.1	6.7	2.0	3.6
7	16.2	20.4	9.9	10.2	3.4	2.7
mean	12.8	15.7	7.4	8.8	5.5	3.3
SD	2.3	5.3	5.8	2.9	3.5	0.9
error	0.9	2.0	2.2	1.1	1.3	0.3

Note: F_p_: plasma flow [mL/100 mL/min]; V_p_: plasma volume [%]; PS: permeability-area surface product [mL/100 mL/min]; ^a^baseline; ^b^follow-up.

**Table 2:** Individual values of functional parameters of tumor microcirculation in the control group at baseline and follow-up.

Animal no.	^a^F_p_	^a^V_p_	^a^PS	^b^F_p_	^b^V_p_	^b^PS
8	19.2	17.2	2.4	26.2	30.8	8.3
9	14.0	12.0	8.1	24.2	12.1	9.0
10	11.8	7.5	6.6	17.3	8.1	7.3
11	10.5	12.4	1.9	22.2	21.3	9.7
12	12.9	14.0	2.8	29.8	25.7	7.8
13	25.5	17.4	11.1	24.2	12.7	16.3
14	12.5	13.4	5.3	22.7	12.1	5.3
15	13.2	18.7	0.5	20.8	9.1	8.5
mean	15.0	14.1	4.8	23.4	16.5	9.0
SD	5.0	3.6	3.6	3.7	8.4	3.2
error	1.8	1.3	1.3	1.3	3.0	1.1

Note: F_p_: plasma flow [mL/100 mL/min]; V_p_: plasma volume [%]; PS: permeability-area surface product [mL/100 mL/min]; ^a^baseline; ^b^follow-up.

### Immunohistochemistry

**Figure 5:**
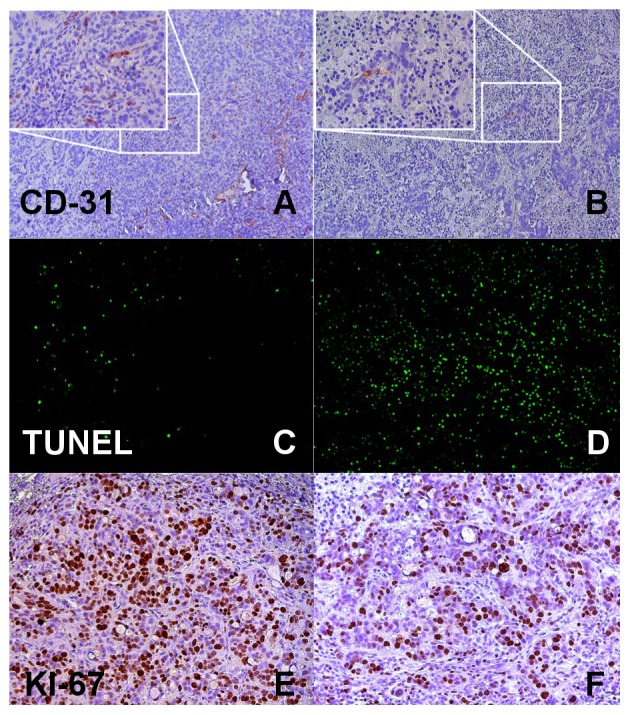
Immunohistochemical analysis. Representative immunohistochemical stainings for microvascular density (CD-31, A and B), apoptosis (TUNEL, C and D), and proliferation (Ki-67, E and F) in the therapy (right) and control group (left). Note the lower number of CD-31 and Ki-67 positive cells (stained in brown, B and F) and the higher number of cells positive for TUNEL (stained in fluorescent green, D) in the therapy group.

Tumor microvascular density (MVD) quantified by CD-31 staining was significantly reduced following regorafenib therapy (therapy group 48±10; control group 112±25; p<0.05). Less proliferating cells (Ki-67; therapy group 4277±1017; control group 4841±1593; p>0.05) and more apoptotic cells (TUNEL; therapy group 11844±2927; control group 5097±3463; p<0.05) were observed in the therapy group. Individual values for these parameters are displayed in Table 3. Fig. 5 shows representative immunohistochemical images.

**Table 3:** Individual values of immunohistochemical markers for microvascular density (CD-31), apoptosis (TUNEL), and proliferation (Ki-67) in the therapy (top) and control group (bottom).

Animal no.	CD-31	TUNEL	Ki-67
1	36	14093	3770
2	55	8253	3796
3	52	9885	3320
4	57	10621	3860
5	59	17100	3707
6	41	11807	5776
7	34	11152	5709
**mean**	48	11844	4277
**SD**	10	2927	1017
**error**	4	1106	384
8	93	1442	7869
9	74	9612	5702
10	86	3845	4958
11	143	2564	3747
12	138	2317	3372
13	117	5313	3201
14	126	4728	5884
15	126	10953	3997
**mean**	113	5097	4841
**SD**	25	3463	1593
**error**	9	1224	563

### Correlation between DCE-CT and Immunohistochemistry

Tumor F_p_ showed a significant positive correlation with tumor MVD (r = 0.84; p<0.05) and a significant negative correlation with tumor cell apoptosis (r = −0.66; p<0.05). Tumor V_p_ showed a significant positive correlation with tumor MVD (r = 0.66; p<0.05) and a significant negative correlation with apoptosis (r = −0.71; p<0.05). Table 4 presents detailed correlation data.

**Table 4:** Linear correlation coefficients (Pearson’s r) for correlation between tumor microcirculation and immunohistochemical parameters (IHC) in the therapy group.

Perfusion/IHC	R	p-value
F_p_/Ki67	0.19	>0.05
**F_p_/TUNEL**	−**0.66**	**<0.05**
**F_p_/CD-31**	**0.84**	**<0.05**
V_p_/Ki67	0.25	>0.05
**V_p_/TUNEL**	−**0.71**	**<0.05**
**V_p_/CD-31**	**0.66**	**<0.05**
PS/Ki67	−0.04	>0.05
PS/TUNEL	−0.11	>0.05
PS/CD31	0.71	>0.05

Note: Statistically significant correlations are printed bold.

## Discussion

In the US, regorafenib has most recently been approved by the FDA for patients with advanced colon carcinoma [21]. In the present study, the potential of DCE-CT functional imaging biomarkers for the non-invasive monitoring of regorafenib monotherapy was investigated in an experimental model of colon carcinoma in rats. Results were validated by multiparametric immunohistochemistry. DCE-CT was used to detect significant changes in tumor microcirculation due to the anti-angiogenic and anti-proliferative effects of regorafenib. Microcirculatory changes showed significant correlations with immunohistochemical results, supporting the hypothesis that non-invasive DCE-CT would be clinically applicable to generate imaging biomarkers for monitoring early changes in tumor physiology under molecular therapy *in vivo*. Correspondingly, immunohistochemical staining revealed a significant reduction of tumor microvascular density (CD-31) and a significant increase in apoptosis (TUNEL) under regorafenib treatment. In addition, TUNEL staining showed a significant increase in apoptosis in the treatment group. These results indicate that regorafenib exhibits pro-apoptotic effects on experimental colon carcinoma xenografts *in vivo*.

### DCE-CT Imaging Biomarkers

Significant reduction of plasma volume and almost significant reduction of plasma flow quantified by DCE-CT was observed in the therapy group. While endothelial permeability increased significantly in the control group, only a non-significant decrease was found in the therapy group. Results are in accord with a previously published preclinical study in which regorafenib and the angiogenesis inhibitor DC101 were evaluated in an orthotopic colon carcinoma model in mice [24]. Compared to DC101, higher suppression of tumor microcirculation as monitored by DCE-MRI was achieved using regorafenib. Additionally, no liver metastases were seen in regorafenib-treated animals. In DC101-treated animals, metastasis rate was only reduced by 33% compared to the vehicle group. This finding suggests an additional anti-metastatic effect of regorafenib, which needs to be confirmed in future trials. Moreover, results are in accordance with studies which showed that DCE-CT may be used to monitor microvascular changes induced by anti-angiogenic therapy in different tumor models [10], [17], [25]. Tai et al. examined the immediate effects of vandetanib in a colon carcinoma xenograft model in nude rats [25]. The authors observed a significant decrease of tumor blood flow and blood volume in the therapy group with a simultaneous increase of both parameters in the control group. It was concluded that DCE-CT is able to monitor immediate microcirculatory changes induced by an anti-angiogenic agent, most likely preceding changes of tumor volume, as well as alterations of immunohistochemical parameters. Results from Tai et al. support the finding that functional imaging biomarkers of tumor microcirculation quantified by DCE-CT may be used to quantify response to small molecule oncologic therapies with more sensitivity and with earlier monitoring of morphological changes of the tumor. Clinically, Lind et al. studied changes in tumor perfusion assessed by DCE-CT in 23 non-small cell lung cancer patients treated with a combination of sorafenib and erlotinib over a course of six weeks [16]. An early decrease in tumor perfusion showed significant correlation with tumor regression monitored by RECIST and overall outcome. The trial by Lind et al. demonstrates the feasibility and applicability of DCE-CT in a clinical patient setting and further shows the association of functional imaging biomarkers with traditional tumor therapy evaluation criteria. Nonetheless, this study does not help to understand molecular changes in tumor physiology, as it does not contain any immunohistological validation.

It therefore is preferable that studies investigating DCE-CT in early tumor therapy assessment use immunohistochemical validation in a monotherapy regime, to verify that the decrease in tumor microcirculation, in fact, reflects therapy-induced changes on a cellular basis. In the long term, non-invasive imaging biomarkers assessed by DCE-CT bear the potential for clinicians of distinguishing responders from non-responders during early phases of treatment. Another challenge is the quest for predictive biomarkers to optimize patient selection most likely to benefit from regorafenib treatment [26]. Given the fact that the leading action mechanism of regorafenib is inhibition of angiogenesis [27], one could hypothesize that tumors being relatively hypervascular, as assessed by DCE-CT, show a better response. In a previous clinical trial, it was shown that patients with colorectal carcinoma presenting a high vascular permeability as assessed by DCE-MRI show a better response to neoadjuvant chemoradiotherapy [28]. Imaging biomarkers would be a step toward a more personalized and targeted medical treatment with regard to maximum benefit for the cancer patient, as well as cost-effectiveness in a healthcare environment.

### Immunohistochemical Validation of Imaging Biomarkers

In the regorafenib-treated group, reduction of plasma flow and plasma volume correlated significantly with a decrease in microvascular density, and with an increase in apoptosis of tumor cells. These results are in accord with a previous study in which a one-week regimen of sorafenib in an experimental prostate carcinoma xenograft model significantly reduced tumor perfusion, vascularity, and endothelial permeability with good-to-moderate correlations with an immunohistochemical standard [17].

Our results indicate that DCE-CT may also allow for the non-invasive and timely assessment of early therapy effects of regorafenib in colon carcinoma. However, these preclinical results remain to be validated in clinical studies, and should be correlated to established clinical tumor response evaluation criteria, such as RECIST, progression-free survival, and overall survival.

In the present work, endothelial permeability did not correlate with any of the histological markers studied. This is consistent with other literature data acquired with micromolecular contrast media [17]. It can still be assumed that endothelial permeability is a functional parameter influenced by anti-angiogenic therapy. However, endothelial permeability does not appear to correlate with tumor proliferation, microvascular density or apoptosis. Another potential reason for the lack of correlation between endothelial permeability and immunohistochemical markers might be that a small molecule contrast media was used rather than a macromolecular contrast media. This hypothesis is supported by the results of Raatschen et al. who investigated the efficacy of the angiogenesis inhibitor bevacizumab in an experimental rodent breast cancer xenograft model using DCE-MRI and macromolecular contrast media. The authors reported significant effects of bevacizumab on endothelial permeability [29].

DCE-CT may be an attractive tool for the non-invasive assessment of regorafenib therapy effects, as it is widely available, inexpensive, has a high spatial resolution, and could be integrated into routine CT protocols [12], [17]. Furthermore, DCE-CT bears the potential to be combined with molecular imaging techniques, such as fluorodesoxyglucose positron emission tomography, to gain additional information on tumor metabolism [30]. In the near future, DCE-CT could allow for the timely and non-invasive assessment of early therapy effects of regorafenib in patients. As outlined above, several authors have successfully demonstrated the applicability of contrast-enhanced ultrasound and DCE-MRI for the non-invasive assessment of tumor microcirculation [10]–[13]. It thus should be discussed which imaging modality would be best to monitor early therapy effects of anti-angiogenic treatment regimens. Multislice imaging modalities may be less user dependent with higher interobserver reproducibility, depending on user experience. In a recent study using an experimental glioma model in rats, both DCE-CT with iopromide and DCE-MRI with an intermediate-sized contrast agent (Gadomer-17) were investigated for monitoring early treatment effects 24 hours after administration of regorafenib [31]. The authors concluded that both DCE-CT and DCE-MRI were applicable, with regard to differences in contrast agent vascular distribution profiles and kinetics. Due to the lack of trials directly comparing DCE-CT and -MRI for the monitoring of early anti-angiogenic therapy effects, it still remains unclear which modality might be superior. Advantages of DCE-CT as compared to DCE-MRI may be shorter acquisition time, lower costs, and wider availability.

However, in order to establish functional DCE-CT as an accepted clinical tool for the early assessment of the efficacy of anti-angiogenic therapy, several issues remain to be solved. Acquisition parameters, such as spatial resolution, temporal resolution, and total acquisition time, as well as radiation dose and post-processing of the image data, have to be standardized for use in a clinical setting. In addition, no final agreement has been reached on which perfusion parameters reflect early therapy effects most reliably, depending on the underlying tumor entity, and the organ or body region under investigation.

Our study results were limited in several ways. In the present study, an experimental human colon carcinoma xenograft was investigated in a subcutaneous rat model. Possible differences between human and animal physiology, as well as the heterotopic location of the tumor, may limit the transfer of our results to a clinical setting. Also, only a relatively short treatment interval was investigated, while favorable clinical regorafenib effects in patients are observed after months [21]. Thus, it is possible that we missed the full extent of the effects of regorafenib, as well as possible side effects which may limit the applicability of DCE-CT in later stages of the disease. However, we consider this short follow-up time adequate, as our primary goal was to investigate early effects of regorafenib therapy. For quantitative DCE-CT analysis and selection of ROIs, we used semiquantitative maps (AUC/max) to differentiate between vital tumor tissue and central necrosis. With regard to immunohistochemical work-up, there may be alternative immunohistochemical markers that convey a better understanding of HT-29 tumor physiology, potentially leading to increased validity of our results. Moreover, investigation of additional tumor models may lead to a more profound understanding of regorafenib action mechanism.

In conclusion, a one-week therapy with the multi-tyrosine kinase inhibitor, regorafenib, had significant anti-angiogenic and pro-apoptotic effects on experimental colon carcinoma xenografts in rats. In parallel, DCE-CT enhanced with a clinically available, small molecule CT contrast agent was utilized to detect a significant decrease in tumor perfusion and tumor vascularity with good-to-moderate correlations to an immunohistochemical standard. These non-invasive parameters of tumor microcirculation may find use as functional imaging biomarkers of the efficacy of regorafenib therapy on tumor pathophysiology. Future studies will need to focus on the establishment of clinical DCE-CT functional imaging biomarkers in order to gain wide acceptance as a sensitive and reliable method to safely monitor early response to molecular cancer therapy.
